# COSAP: Comparative Sequencing Analysis Platform

**DOI:** 10.1186/s12859-024-05756-z

**Published:** 2024-03-26

**Authors:** Mehmet Arif Ergun, Omer Cinal, Berkant Bakışlı, Abdullah Asım Emül, Mehmet Baysan

**Affiliations:** Department of Computer Engineering, Istanbul Technical University, 34469 Istanbul, Turkey

**Keywords:** NGS Analysis, Variant classification, Variant annotation, Copy number variation, Microsatellite instability

## Abstract

**Background:**

Recent improvements in sequencing technologies enabled detailed profiling of genomic features. These technologies mostly rely on short reads which are merged and compared to reference genome for variant identification. These operations should be done with computers due to the size and complexity of the data. The need for analysis software resulted in many programs for mapping, variant calling and annotation steps. Currently, most programs are either expensive enterprise software with proprietary code which makes access and verification very difficult or open-access programs that are mostly based on command-line operations without user interfaces and extensive documentation. Moreover, a high level of disagreement is observed among popular mapping and variant calling algorithms in multiple studies, which makes relying on a single algorithm unreliable. User-friendly open-source software tools that offer comparative analysis are an important need considering the growth of sequencing technologies.

**Results:**

Here, we propose Comparative Sequencing Analysis Platform (COSAP), an open-source platform that provides popular sequencing algorithms for SNV, indel, structural variant calling, copy number variation, microsatellite instability and fusion analysis and their annotations. COSAP is packed with a fully functional user-friendly web interface and a backend server which allows full independent deployment for both individual and institutional scales. COSAP is developed as a workflow management system and designed to enhance cooperation among scientists with different backgrounds. It is publicly available at https://cosap.bio and https://github.com/MBaysanLab/cosap/. The source code of the frontend and backend services can be found at https://github.com/MBaysanLab/cosap-webapi/ and https://github.com/MBaysanLab/cosap_frontend/ respectively. All services are packed as Docker containers as well. Pipelines that combine algorithms can be customized and new algorithms can be added with minimal coding through modular structure.

**Conclusions:**

COSAP simplifies and speeds up the process of DNA sequencing analyses providing commonly used algorithms for SNV, indel, structural variant calling, copy number variation, microsatellite instability and fusion analysis as well as their annotations. COSAP is packed with a fully functional user-friendly web interface and a backend server which allows full independent deployment for both individual and institutional scales. Standardized implementations of popular algorithms in a modular platform make comparisons much easier to assess the impact of alternative pipelines which is crucial in establishing reproducibility of sequencing analyses.

**Supplementary Information:**

The online version contains supplementary material available at 10.1186/s12859-024-05756-z.

## Background

Sequencing technologies become more accessible as they generate more data in less time for diminishing costs [1]. However, the computational requirements of processing NGS data are much higher than what most clinical facilities and biomedical laboratories have. The level of programming skills required for using mapping, preprocessing and variant calling algorithms efficiently is fairly high [2]. Furthermore, concordance among sequencing algorithms is limited especially for cancer sequencing [2]. There are many sequencing algorithms that can perform well in different settings and choosing the best combination of sequencing algorithms for a dataset is very difficult. It’s been shown that combining multiple algorithms improves performance and ensemble methods are developed along this aim [3, 4]. Despite this improvement, relying on a single combination that would work well for all possible scenarios is impossible considering the heterogeneity of applications in research and clinic.

There have been many previous successful efforts to provide researchers with flexible open source sequencing analysis pipelines and platforms. Galaxy [5] and Terra [6], which are the most commonly used platforms, harbors many of the commonly used algorithms for the analysis of genomics, metagenomics, transcriptomics as well as other omics data. Both platforms allow users to create and share workflows on the workflow hub. All that flexibility brings a steeper learning curve for beginners. Even though it is possible to deploy them locally, they are essentially used as cloud services. This brings a barrier for many users as local local regulations can be restrictive to share data with third parties over the internet. With the advancement of hardware acceleration technology, especially GPU’s, the demand for local handling of the sequencing data is increasing. These platforms are yet to respond to that demand. Moreover, provided variant annotations are very limited despite the platform being under development for years. Sarek [7] is another NGS analysis workflow which is built on Nextflow [8] workflow language. It provides limited number algorithms for the detection and analysis of germline and somatic mutations. Also it does not have a user interface and backend to manage user files and runs. DNAScan [9] and Sequana [10] are other available predefined NGS analysis workflows. They have very limited interfaces just to upload data files and lack a broad range of algorithms for comparative analysis of variants. The Table 1 shows a summary of feature comparison of tools.

**Table 1:** Comparison of features of similar tools

Tool	Scope	Computing environment	User interface features	Hardware acceleration
Sequana	DNA-seq/RNA-seq	Local	Pipeline creation	N/A
DNAScan	DNA-seq	Local	Pipeline creation	N/A
Sarek	DNA-seq/RNA-seq	Cloud/Local	Pipeline creation	N/A
Galaxy	Extensive from genomics to metagenomics	Cloud/Local	Pipeline creation and extensive analysis on output data	N/A
Terra Bio	DNA-seq/RNA-seq	Cloud	Pipeline creation and extensive analysis on output data	GPU Acceleration via Clara Parabricks
Cosap	DNA-seq	Cloud/Local	Pipeline creation and detailed inspection of results and reports	GPU Acceleration via Clara Parabricks

In order to address the above-discussed limitations of the sequence analysis tools, in this paper, we propose Comparative Sequencing Analysis Platform (COSAP) as an alternative analysis platform. COSAP offers multiple options for each step of the analysis pipeline and allows users to compare the effect of these choices by producing multiple VCF files each representing a particular combination. COSAP simplifies planning and running sequencing pipelines both for skilled developers and non-technical users. COSAP can run locally or be deployed on a server to be accessed by many users from their local devices via a web interface. Users can create desired pipelines from pre-installed algorithms for mapping/aligning, pre-processing, variant calling and annotation. COSAP first creates a template file that contains all of the information that is needed to execute every step. Execution can be done in two ways. First, most users can utilize COSAP’s user-friendly web interface to upload their files and run analysis by selecting dropdown menus, monitor the progress of running analyses as well as visualize the results. Second, advanced users can take this a step further and use COSAP’s underlying Python API. This API allows the construction of more complex pipelines. New pipeline steps can be introduced by coding them into COSAP’s codebase with a streamlined process due to COSAP’s intuitive software design. Since constructing and running pipelines are decoupled, template files can be created from the API and then run from UI and vice versa.

## Available tools

### Fastq preprocessing and short read mapping

Preprocessing and quality checking of raw reads is the first step in most NGS analysis. Fastp [11] which is an all-in-one tool for fastq file quality control and filtering is used for these tasks in COSAP. For the short read mapping, BWA [12] and Bowtie2 [13] are the most commonly used tools and are available in COSAP. The newer and faster version of BWA is also included [14].

### Aligned read preprocessing and filtering

There are several BAM preprocessing steps before variant calling recommended in the GATK Best Practices guidelines [15]. COSAP utilizes GATK4 [16] and Samtools [17] to handle these steps. Sort, index and mpileup commands from Samtools and, all tools from GATK are currently available.

### SNV, Indel and structural variant discovery

Variant calling is one of the most extensively studied areas in the NGS research and a whole raft of variant callers are developed and utilized in the literature. Currently, COSAP supports 11 variant callers and 1 deep learning based variant refinement tool [18]. These callers are HaplotypeCaller [19], Varscan2 [20], Strelka2 [21], and DeepVariant [22] for germline samples. The included somatic variant callers are Mutect2 [19], Varscan2 [20], Varnet [23], MuSe [24], VarDict [25], Octopus [26], SomaticSniper [27]. The structural variants are called with Manta [28].

### Variant annotation

The variant annotation is an essential step to extract meaningful information from the variant sets. Variants are annotated by using many tools depending on the sample type, variant type and the need of the researcher. COSAP supports Ensembl VEP [29], SnpEFF [30], Annovar [31] for functional annotations of SNV’s. The AnnotSV [32] is used to annotate structural variants and ClassifyCNV [33] is the tool of choice for annotating copy number variations. Germline and somatic variants are automatically classified according to ACMG/AMP [34, 35]guidelines by InterVAR [36] and CancerVAR [37] accordingly. GenomeNexus [38] which is a tool to annotate variants from multiple sources is also available with COSAP.

### Implementation

There is a plethora of NGS algorithms available for preprocessing, mapping, alignment, variant calling and annotation. Unfortunately, many of these algorithms are not well-documented and new methods or algorithms are developed that fulfills various needs regularly. Therefore we developed a fully customizable and open source platform and integrated the popular algorithms into this platform. This level of customizability is achieved by several design decisions.

COSAP has been built around the principle of modularity of pipeline steps. Every step is abstracted into a Python class where the dependencies and algorithms involved in that step are all included. These Python classes work in sync with whichever algorithm or algorithms they need to call and only exit once the processes are either finalized or an exception is encountered. The encapsulation of all pipeline steps with identical method signatures means that can move blocks around and build complex networks of algorithms without knowing the internal workings, like building Lego blocks.

**Fig. 1:**
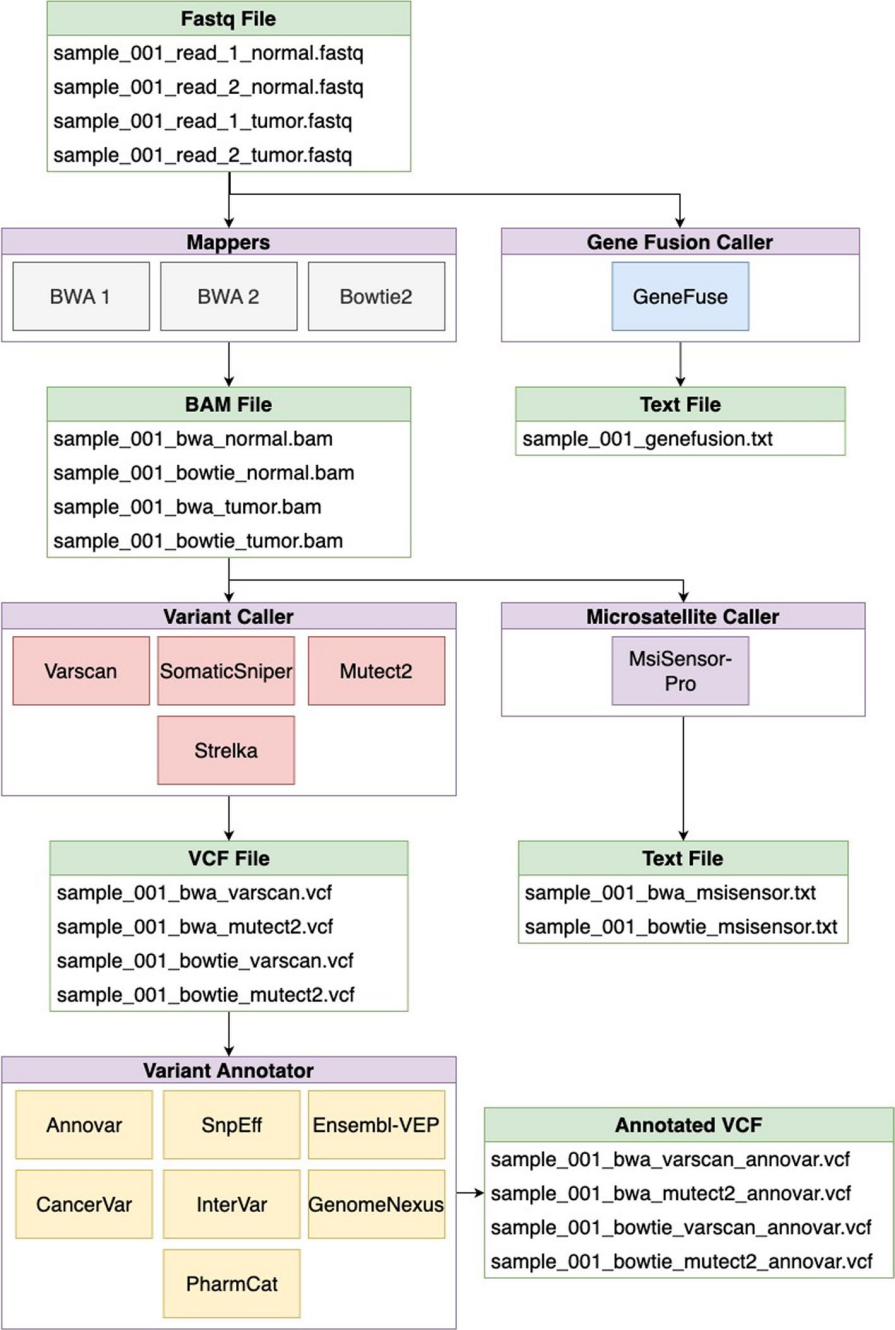
Predefined pipeline steps have multiple algorithm/tool choices. The input and output of each step must be a list of files. The file names are for humans to understand, and steps know which file to read from a config file

COSAP comes with the most popular algorithms used around the built-in NGS pipelines. This way for most use cases no new pipeline needs to be defined. These predefined steps are mainly targeted at somatic and germline pipelines. COSAP also includes predefined libraries for exome and genome panels. Custom libraries can be added in any location as long as their path is known to COSAP. The available tools, their outputs and general workflow of the COSAP pipeline is shown in Fig. 1.

**Fig. 2:**
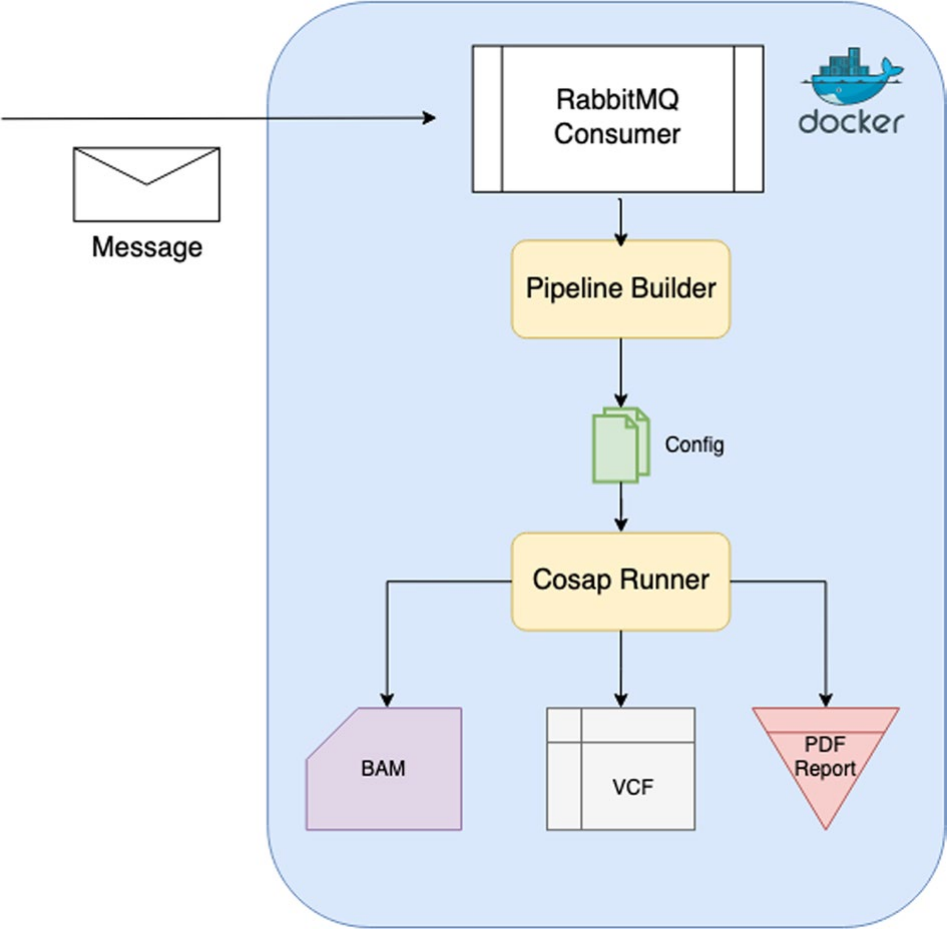
COSAP Docker container as a celery worker which consumes pipeline messages from the backend application

We also implemented fully functional web and backend applications as part of COSAP. The web application communicates with the backend via REST API. COSAP Docker containers are bound to the same storage with the backend and can work as celery workers which consume messages from the backend application. There may be multiple workers on the network which brings scalability to the platform. The message queuing architecture is shown in Fig. 2.

### Software design

COSAP is a Python based modular tool that is designed to create NGS pipelines. Each pipeline step (aligning, variant calling, annotation, etc.) is written in its own classes following an abstract format. This abstraction allows each step to be created and run separately from each other. Custom steps can be written following the same abstract pattern. This means pipelines with any length and tool can be created like a graph. COSAP analysis consists of two major parts.

The first part contains the API that allows the user to define input files and pipeline steps, and then form one or more pipelines using these steps. The output of this creation is a pipeline state file in JSON format. This file contains all the information required to execute each step. It includes all input and inflight file names, location of algorithms and reference files, parameters for algorithms, timestamps and version information. The API has classes for complicated parameters to be passed along to algorithms and some predefined classes that can create most commonly used pipelines with a single or few lines of code.

The second part handles the running of the pipelines. This accepts a pipeline state file which is created by the first step automatically or manually edited by hand. There are two essential ways that a pipeline state file can be run. The legacy method runs each pipeline step defined in the state file in a sequence. The second method uses Snakemake [39] to optimize the resource usage (CPU and memory) by running steps in parallel if their requirements are satisfied. These requirements are essentially sufficient resources being available for the step to be run and files needed by the step are finalized or ready. Explained in detail in the performance section.

The decoupling of these two parts allows them to be assigned to different systems. The creation of pipeline states is not a resource heavy operation and can be handled by a smaller server whereas the second part (execution) can be split into clusters.

## Results

### Performance

Executing NGS pipelines may require large amounts of processing time and optimizing pipelines to leverage the full potential of modern hardware is extensively studied in the literature. Currently, hardware based accelerations such as NVIDIA Clara Parabrics [40] and Illumina Dragen [41] offer the best performance in terms of speed. These claim up to 80 × speed gain over the baseline GATK [16] tools. They also don't make a concession of variant calling accuracy as DRAGEN short-read call set was the top performer in PrecisionFDAv2 [42] as well as other studies [43] and Parabricks performed comparably in the benchmark study of Franke et al. [44]. The main of this approach is the requirement of specialized hardware such as FPGA and GPU which are inaccessible to many users. Software optimizations mostly aim for algorithmic improvements and better utilization of the hardware by adapting modern dataflow architectures and hyperthreading and may reach up to 16 × speed gain [45–47]. The problem with that approach is the modifications on the tools’ original code bases in order to make them compatible with other frameworks. These modifications are both error prone and forces users to stick to a specific version. In a previous study, Ahmad et al. [47] suggested that parallelizing GATK over RamDisk performed slightly worse than modified tools achieving ~ 3.5 × speed. Because of these reasons, we decided to utilize shared memory of Linux systems for in memory parallelization.

One of the most time consuming operations is the serialization and deserialization process, especially writing and reading files from the disks between pipeline steps. COSAP can be configured to use previously mentioned shared memory for these intermediate files. This means pipeline steps still need to perform serialization but the in memory read write speeds are incomparably higher than of reading from a disk. This kind of approach has two caveats. First, the memory requirement for naive algorithms is already high. When this is used along with pipeline step parallelization, the memory requirement will be a lot higher. The second issue is in case of a system failure, the memory (hot storage) may lose the data and pipeline steps would have to be run again. COSAP circumvents the second issue by writing the file into disk in a parallel thread. This momentarily slows down the computational capability of the system as the default compression and serialization algorithms used, usually heavily relies on CPU. However, compared to the alternative this hold up is negligible.

**Fig. 3:**
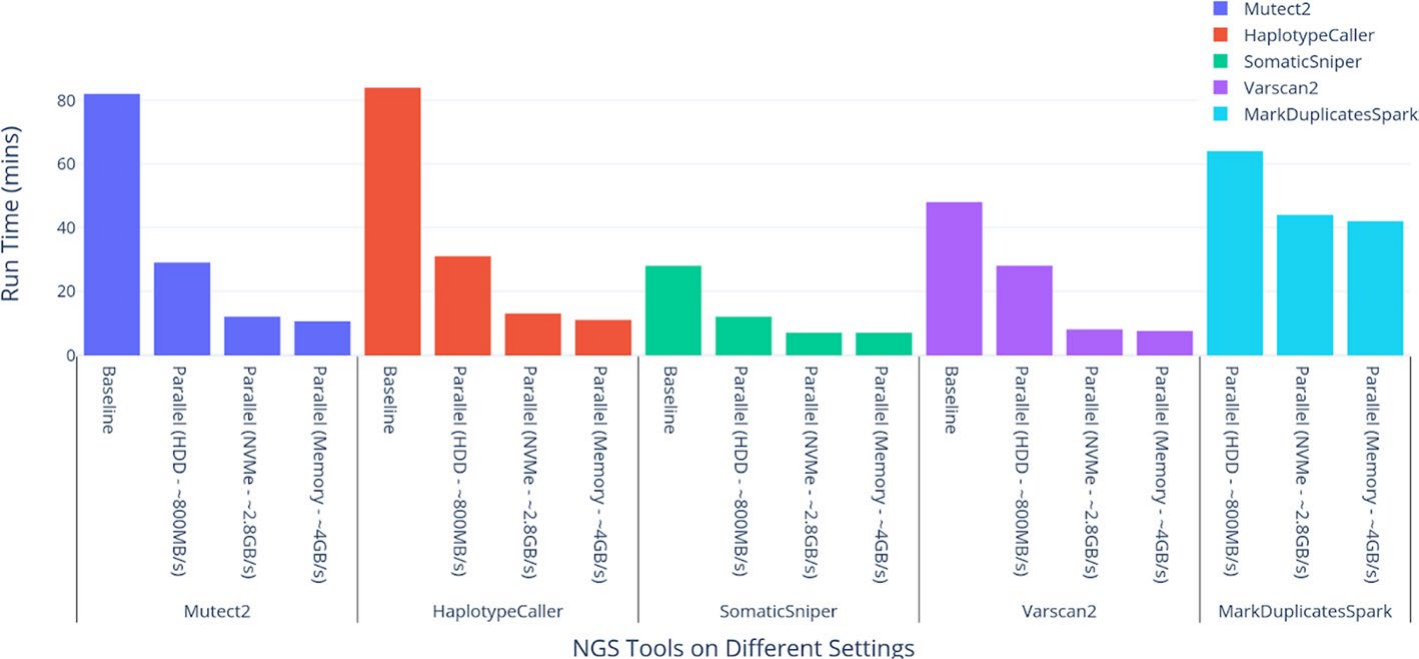
Performance of parallelized versions of the tools on different disk speed settings in comparison with the baselines

The scatter–gather method utilized in the COSAP achieves significant performance gains even when run on slow disk (~ 800 MB/s) (Fig. 3). The speed increases by a factor of 2 when the COSAP DNA pipeline runs on a NVMe drive (~ 2.8 GB/s). Ramdisk performs the best in all parallelized tools with up to 8 × speed gain compared to default implementation of the tools. COSAP currently accommodates parallelized versions of Mutect2, HaplotypeCaller from GATK toolset as well as Varscan2 [20] and SomaticSniper [27]. Other available variant callers have built-in multithreading support. The performance benchmarks are performed on an Intel Xeon E5-2680 v4 chip with 128 GB of memory using WES data from Sequencing Quality Control 2 [48] datasets with accession numbers of SRR7890850 and SRR7890851.

### Python API

Python is a widely used programming language that can tackle both high and low level problems at the cost of performance in most cases. Since many of the applications and algorithms popular in NGS pipeline are coded in higher performing languages, Python language can be used to wrap around these libraries and only used for an adapter layer for enabling research. COSAP’s Python API allows Keras-like networks of NGS pipeline steps to be created. Custom or predefined pipeline step classes can be connected to create these networks.

### Comparing pipelines and benchmarking

**Fig. 4:**
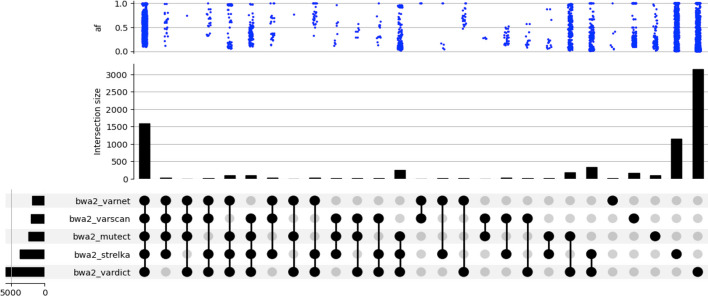
Upset plot depicts the intersection between variant sets of several variant callers, and variant allele frequency distribution of each intersection

COSAP’s comparison and benchmark module help users to analyze their pipelines deeper and finetune accordingly. Users may have different motivations to make comparison and benchmarking analyses such as ensuring the instrument is working as expected or targeted methods capture all of the variants in clinically relevant regions, as Olson et al. argues [49]. They also present an “Overview first, zoom and filter, details on demand” framework for variant visualizations. COSAP’s comparison module follows a similar path where it visualizes the overall pipeline intersection and similarity which gives general understanding. It also gives users an option to draw double and triple venn diagrams of the pipelines or tools of their choice. Figure 4 shows an example comparison of variant callers on previously described seqc2 WES data.

**Fig. 5:**
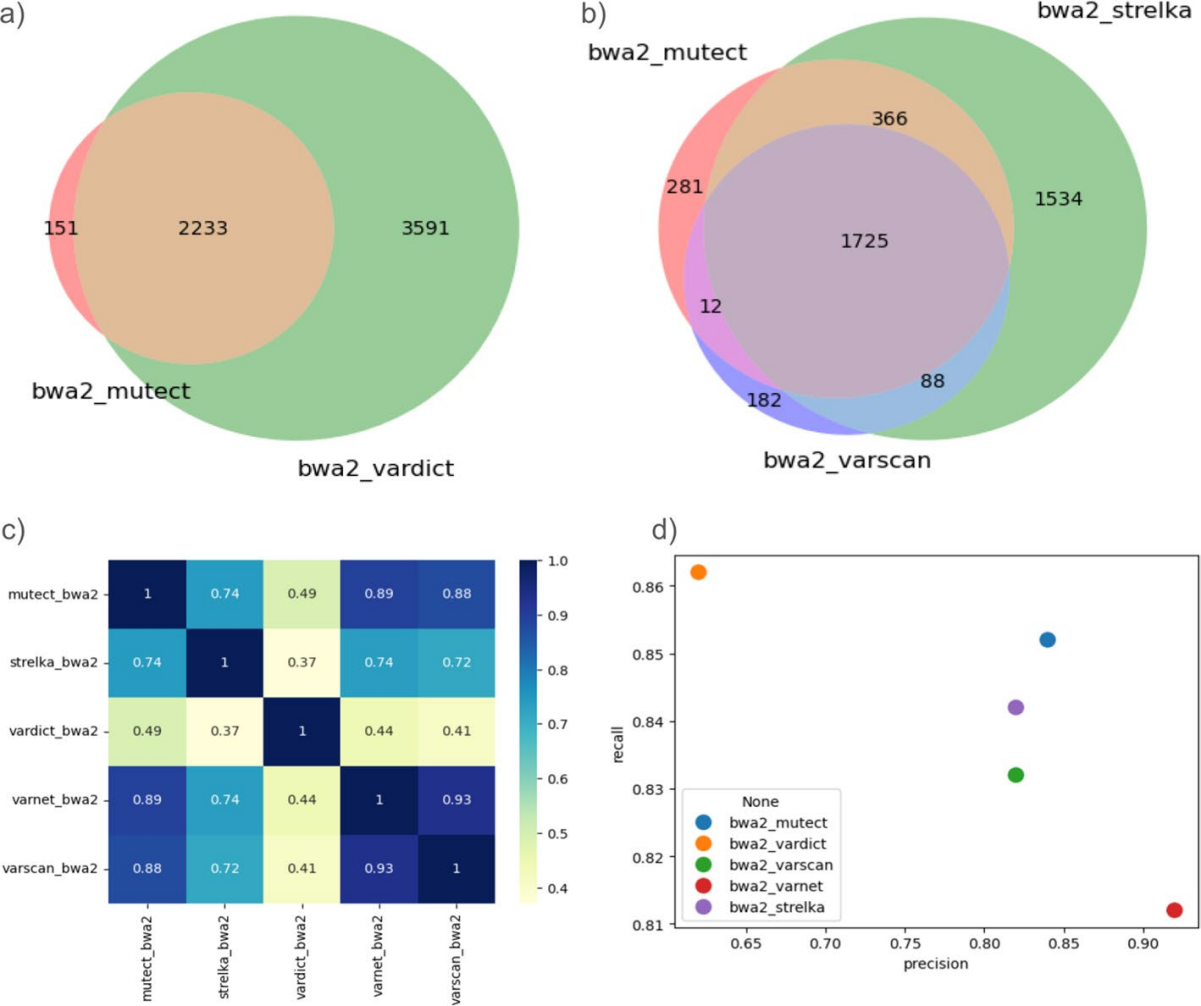
**a** Double venn diagram of chosen variant callers. **b** Triple venn diagram of chosen variant callers. **c** Jaccard similarities of each variant caller. **d** Precision and recall plot when ground truth set is available

Depending on availability of the baseline variant set, COSAP can calculate precision and recall values to assess performance of each pipeline Fig. 5d. This option allows users to pick the best performing combination depending on their sensitivity and specificity needs. When a genome stratification bed file is supplied, the comparison and benchmarking module creates two of each graph enabling the user to see the effect of filtering on intersection and precision/recall values.

Lastly, the comparison module can create bed files for any intersection including TP, TN, FP and FN sets and load them into IGV along with vcf tracks for detailed inspection of variants which might be needed especially for indels and structural variants (Additional file 1: Fig. 1).

### User interface

The user interface of COSAP is implemented as a web application using ReactJS and MaterialUI design components and hosted at cosap.bio/portal. Although it is suggested, users can discover the COSAP web application and utilize it without creating an account. It should be noted that the main purpose of cosap.bio is to demonstrate strengths of the application and when the demand is high there could be long waiting times for jobs to start. The interface provides an easy way of creating projects with the available tools and presents workflow results in an understandable way. With the help of backend services which are powered by Django-Python, users can inspect the results broadly and deeply, modify their results and save them for future analyses. The user interface is also packed with igvJS [50] to help users to visually inspect variants. The main page which displays available services and latest actions is depicted in Fig. 4. On this page, the user can select the analysis type and navigate through other pages.

After selecting the analysis type, the user is directed to the project creation page where the name of the project, required files and desired algorithms are submitted. An example somatic project creation page is shown in Fig. 5. This page slightly changes depending on the required files and algorithms by analysis types.

**Fig. 6:**
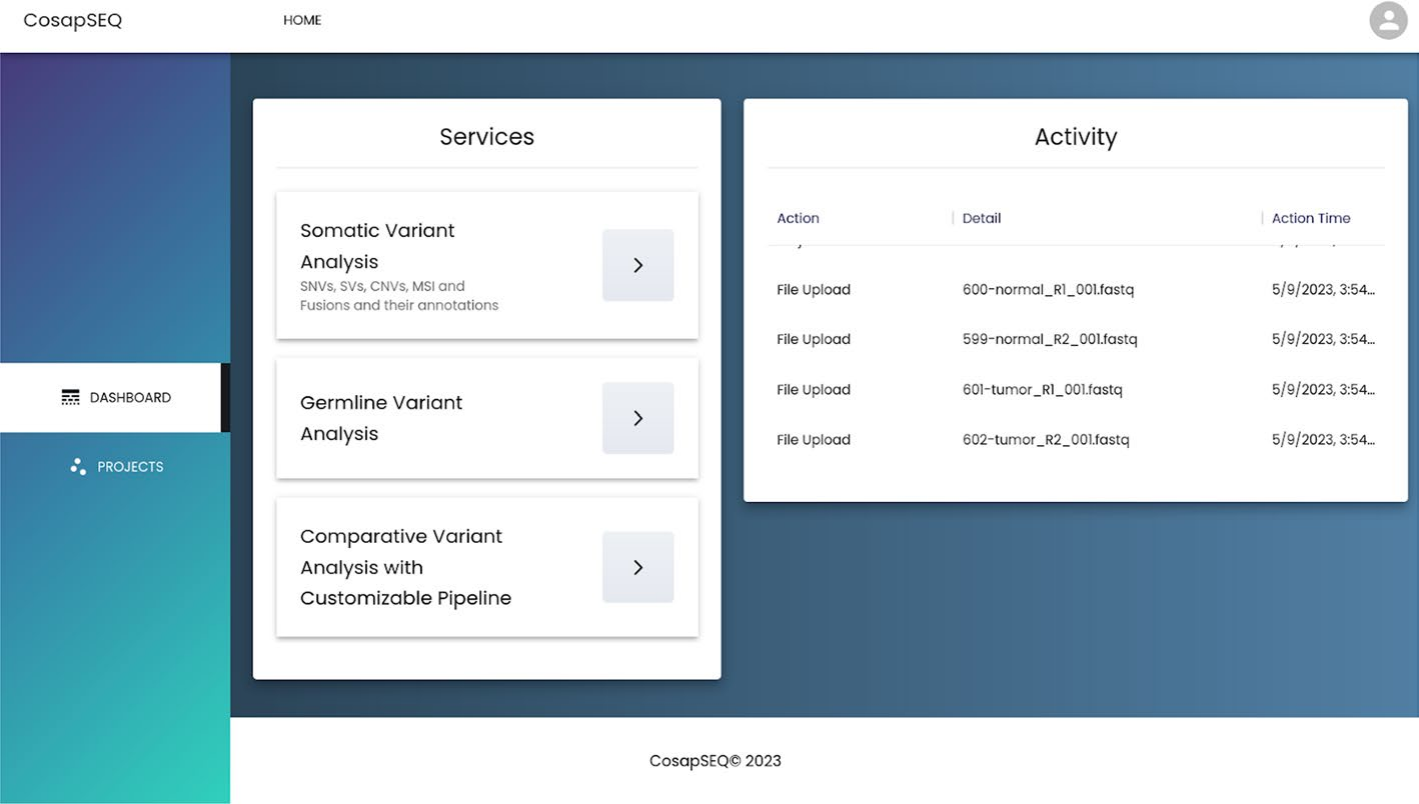
Main page of the web application where users choose the analysis they want to perform and see their recent activity

On the project listing page which is shown in Fig. 6, users can track status of projects and see their details. Navigation to the project creation page is also possible.

**Fig. 7:**
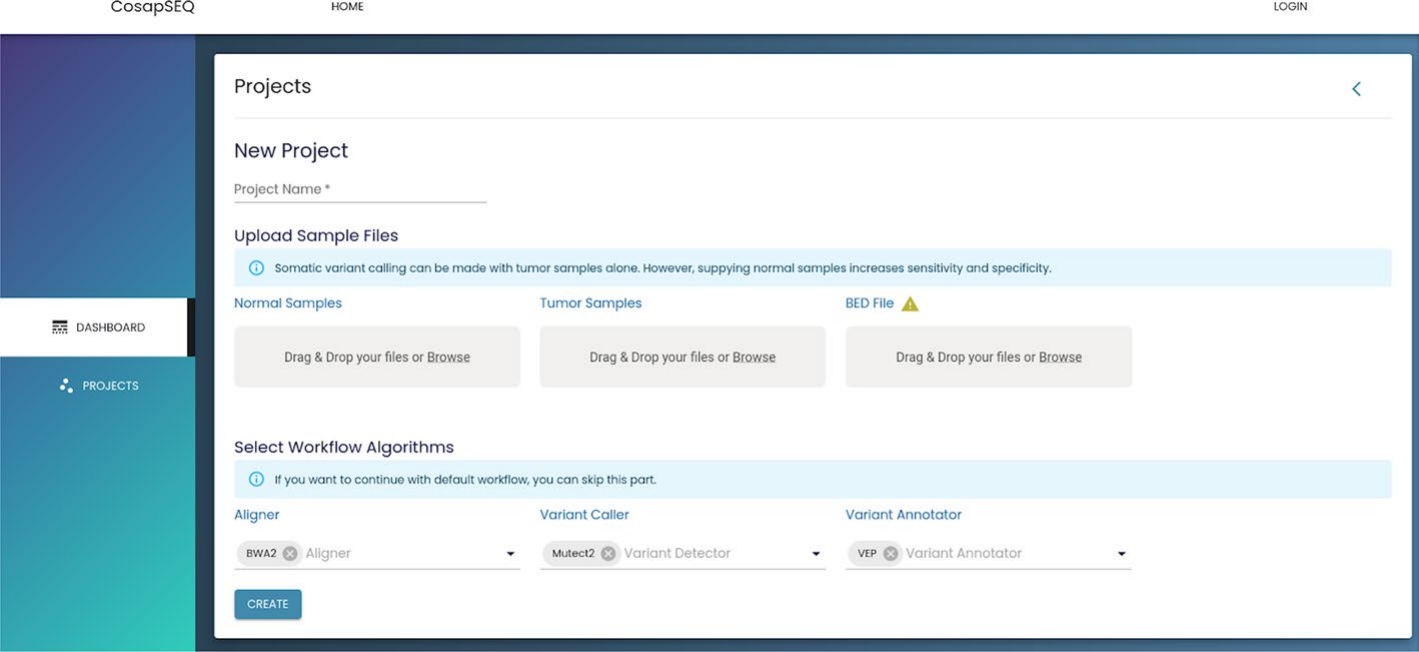
Project creation interface to create projects with input files and desired algorithms

**Fig. 8:**
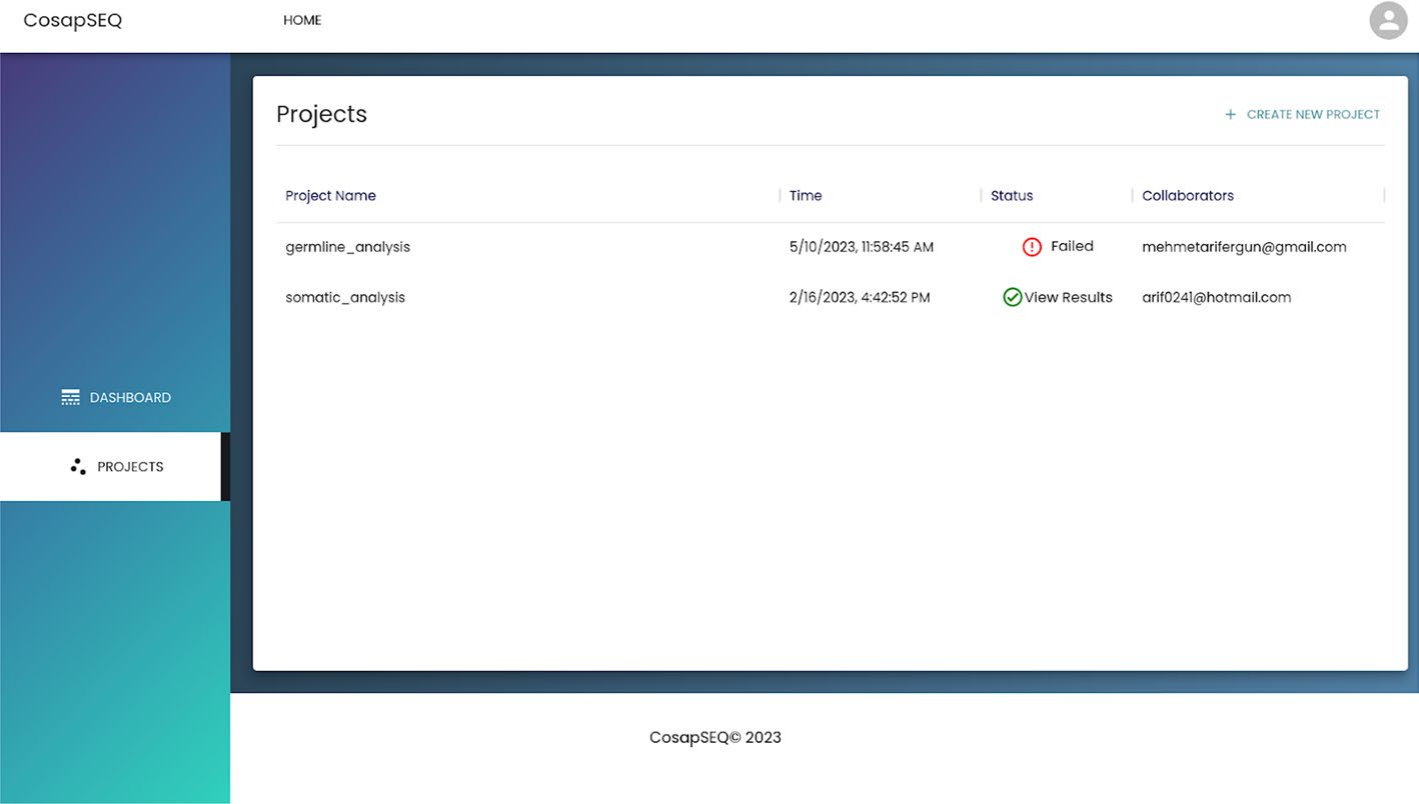
Interface to track status of projects and manage them

**Fig. 9:**
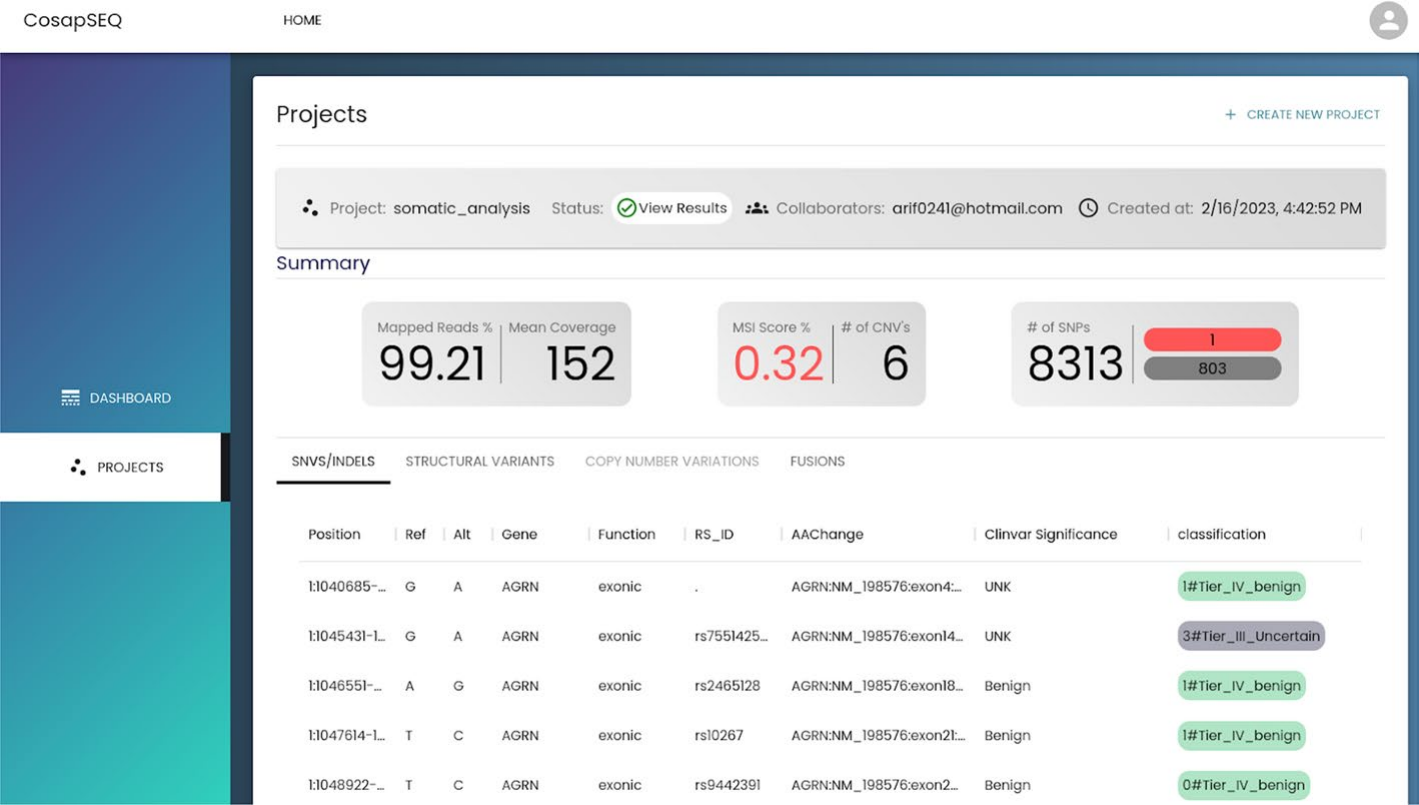
Results page where basic stats of the run is displayed alongside with the detailed variant descriptions and classifications

**Fig. 10:**
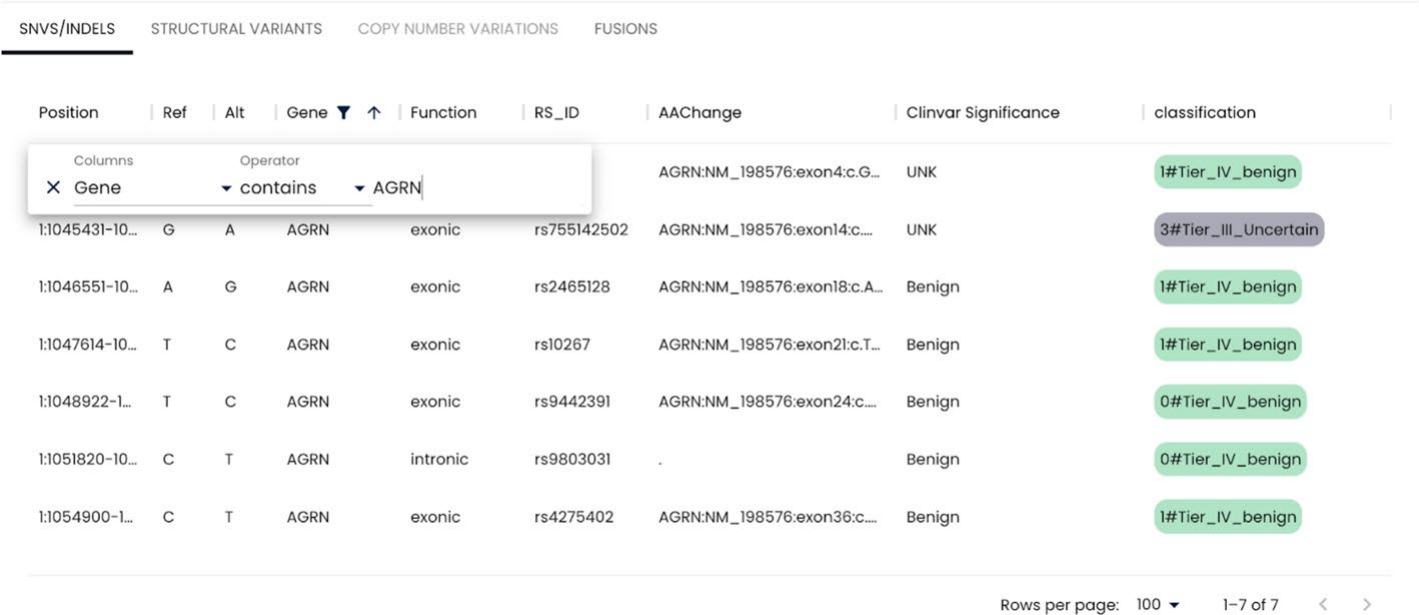
Variant filtering example

Once the project run is finished, the user can navigate to the project results page on Fig. 7. On this page, a summary of results which includes quality control statistics, number of variants and MSI score is shown on top of the page. All the SNVs, INDELs, SVs, CNVs are listed on the page which allows users to filter the variants based on all available annotations. An example filter is shown in Fig. 8 (Figs. 9 and 10).

## Conclusions

COSAP enables technical and non-technical scientists to sequence and analyze DNA by predefined pipelines and with an easy to use UI. Among many other pipeline and sequencing platforms COSAP stands out with availability of comparative analyses, ease of use via the web UI, Python API and, reproducibility via Docker containers and pipeline config files. These design choices and good software practices remove obstacles that come with a plethora of algorithms that are loosely meant to fit together. Automatic generation of command line arguments completely eliminates human errors such as overwriting existing results or running the same experiment twice.

One of COSAP's strengths lies in its software design. COSAP can be conveniently extended with future algorithms which means it will be very difficult for COSAP to be outdated. DNA sequencing requires a lot of computational power which is expensive and unaffordable for most research institutes. We have designed COSAP to be deployable to every system possible with Docker containers. The container can be configured to be run on a laptop or on a cluster of computers with a large pool of resources. Multi-container setup controlled by Celery allows multiple users to run sequences of jobs or a huge number of jobs to be queued and run automatically over an extended period of time. This can all be managed from a simple web app with very low resource requirements.

As the area of genomics is in its infancy, there are a lot of algorithms with eccentric configurations. COSAP handling the execution of all algorithms means each algorithm's parameters, logging and error handling needs to be done for each algorithm. These algorithms have a huge range of methods on these problems varying from logging every debug, info, warning and error to giving successful run results for failed runs. Some algorithms can even cause segmentation faults. As in its current state COSAP can’t handle these types of problems. Another technical difficulty is to configure a cluster of computers to run COSAP containers. There are many solutions for distributed systems and supporting all of them is an impossible task. Therefore, distributed systems and multi-container setups require technical know-how.

In the future, addressing the problems mentioned above is a priority. Even though logging and exception handling is a ceaseless hassle, it can be improved to make debugging more comfortable. Some examples regarding cloud setup aimed at helping configuring multi-container setups are also in the works. Along with examples of infrastructure-as-a-code will be provided in order to make cloud deployment as painless and quick as possible.

### Availability and requirements

Project name: COSAP.

Project home page: https://github.com/MBaysanLab/cosap

Operating system(s): Linux or Other via Docker.

Programming language: Python, JavaScript.

Other requirements: GATK Grch38 bundle and any other database access.

License: MIT.

Any restrictions to use by non-academics: None.

### Supplementary Information

**Additional file 1.** The additional file shows an example IGV view with comparison tracks are loaded along with vcf files.

## Data Availability

COSAP repository access: https://github.com/MBaysanLab/cosap, https://github.com/MBaysanLab/cosap-webapi, https://github.com/MBaysanLab/cosap_frontend Dataset access: WES data from Sequencing Quality Control 2 datasets with accession numbers of SRR7890850 and SRR7890851. The paper of the dataset is 10.1038/s41587-021-00993-6.

## Abbreviations

COSAP: Comparative Sequencing Analysis Platform
NGS: Next generation sequencing
SNV: Single nucleotide variant
SV: Structural variant
CNV: Copy number variation
