# Supplementary figures and images for: COSAP: Comparative Sequencing Analysis Platform
Source: BMC Bioinformatics. 2024 Mar 26;25:130. doi: 10.1186/s12859-024-05756-z (PMC10967217; doi:10.1186/s12859-024-05756-z)

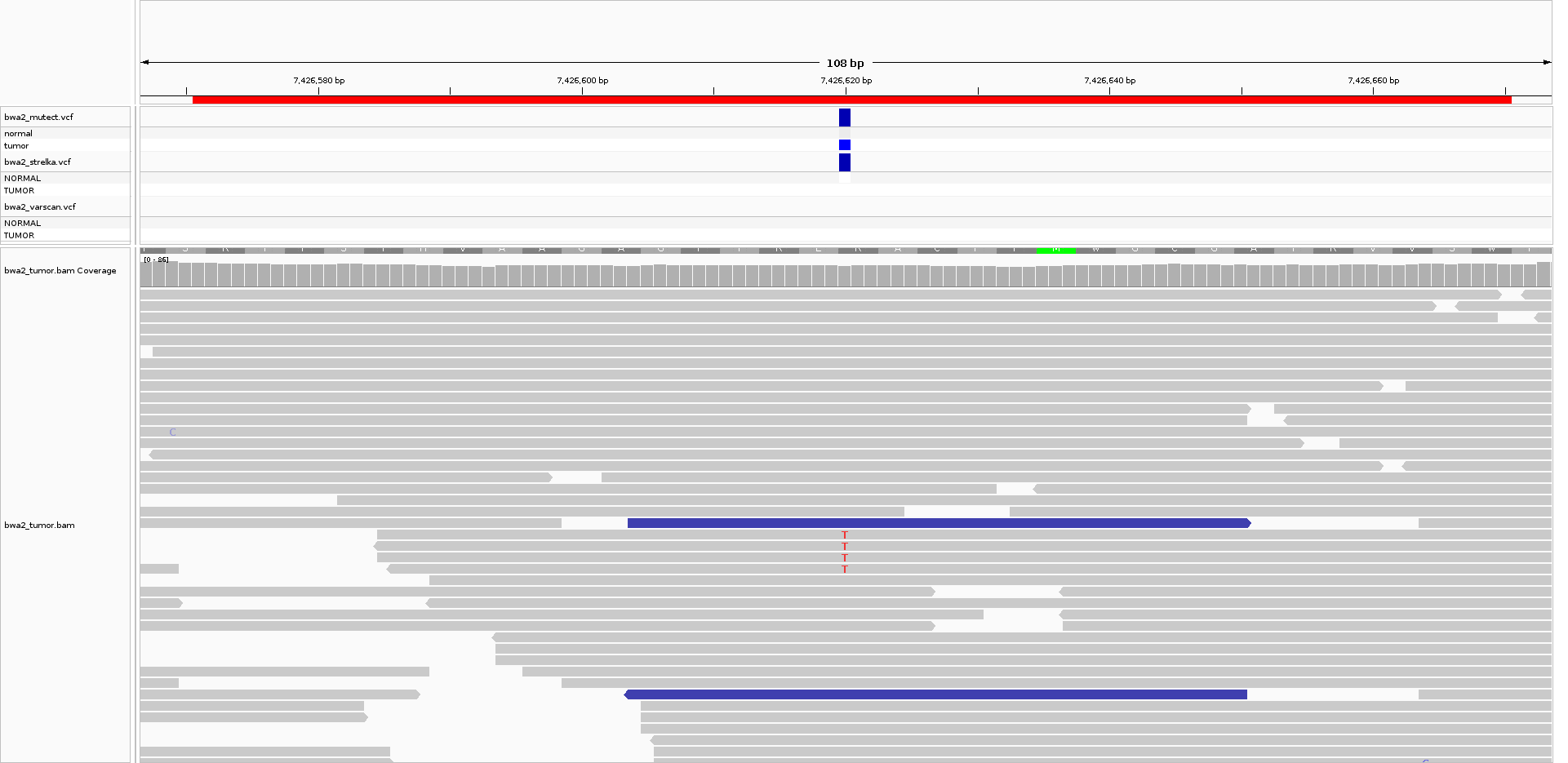

Supplement: Supplementary file 1 — Additional file 1. The additional file shows an example IGV view with comparison tracks are loaded along with vcf files. [file 12859_2024_5756_MOESM1_ESM.png]
